# High‐Temperature Persistent Luminescence and Anti‐Thermal Quenching in LiGa_5_O_8_ by Trap Engineering

**DOI:** 10.1002/advs.76549

**Published:** 2026-07-11

**Authors:** Yuchen Wang, Yilong Fan, Jianhua Liu, Jin Zhang, Dingling Xiong, Tengxiang Long, Lina Su, Chao Sun, Hao Song, Pengpeng Dai, Jingui Duan, Ling Huang

**Affiliations:** ^1^ State Key Laboratory of Chemistry and Utilization of Carbon‐Based Energy Resources College of Chemistry Xinjiang University Urumqi China; ^2^ School of Chemistry and Molecular Engineering Nanjing Tech University Nanjing China; ^3^ Department of Physics and Electronic Engineering & School of Chemistry and Chemical Engineering Key Laboratory for Mineral Luminescence and Microstructure of Autonomous Region Xinjiang Normal University Urumqi China

**Keywords:** anti‐thermal‐quenching, defect engineering, high‐temperature persistent luminescence, LiGa_5_O_8_ phosphors

## Abstract

High‐temperature luminescent materials are essential for applications in displays, information storage, anti‐counterfeiting, and extreme‐environment imaging, yet it remains highly challenging to simultaneously realize anti‐ thermal quenching photoluminescence (anti‐TQ PL) and high‐temperature persistent luminescence (HT‐PersL) under elevated temperatures. In this work, we demonstrate a defect‐engineering strategy based on synergistic Cu^2+^ and Zn^2+^ co‐doping in LiGa_5_O_8_, which reconstructs the deep trap hierarchy. The resulting thermally robust deep trap network enables efficient charge‐carrier storage and thermally activated release of carriers at high temperatures. Consequently, LiGa_5_O_8_:Cu^2+^, Zn^2+^ exhibits both significant anti‐TQ PL and enhanced HT‐PersL, displaying a temperature‐dependent chromaticity evolution from blue to red. This study provides an effective strategy for synergistically controlling multicolor anti‐TQ PL and HT‐PersL in a single material system.

## Graphical abstract

Cu/Zn co‐doping creates deep and thermally stable traps in LiGa_5_O_8_, enabling enhanced high‐temperature persistent luminescence, anti‐thermal‐quenching emission, and dynamic blue‐to‐red color evolution.

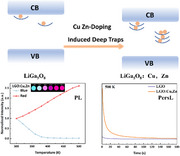

## 1 Introduction

High‐temperature imaging and extreme‐environment applications such as displays and high‐power LED lighting require luminescent materials with stable emission at elevated temperatures [1, 2, 3]. However, most conventional phosphors suffer from severe TQ because elevated temperatures facilitate multi‐phonon relaxation, thermal ionization of activators, and competitive defect‐mediated recombination [4, 5, 6]. Overcoming TQ therefore requires precise manipulation of charge carrier dynamics, defect chemistry, and energy‐transfer pathways‐tasks that remain challenging for the majority of inorganic hosts. A typical example is PersL materials, which require a hierarchy of traps that can effectively capture charge carriers during excitation and then release them gradually after excitation is stopped [7, 8, 9, 10, 11, 12, 13]. However, the primary challenge lies in the contradiction between the mechanism of PersL and elevated temperatures. The thermal energy at high temperatures causes rapid depletion of charge carriers and concurrently intensifies the TQ effect, leading to a drastic reduction in both the duration and intensity of PersL [14, 15, 16]. To expand the operational temperature range of PersL, considerable efforts have focused on developing HT‐PersL materials by defect engineering, such as CaSr_2_Al_2_O_6_:Tb^3+^ [17], Ca_3_Ga_4_O_9_:Tb^3+^/Zn^2+^ [18], SrGa_2_O_4_:Tb^3+^ [19]. All these results indicated that proper deep traps play a key role in HT‐PersL. Until now, HT‐PersL still presents a significant challenge compared to conventional room‐temperature PersL performance, as the elevated temperature accelerates trap depletion and non‐radiative transitions, severely limiting afterglow duration. Simultaneously, suppressing non‐radiative transitions and achieving anti‐TQ PL under elevated temperatures is equally challenging. Furthermore, achieving both anti‐TQ PL and HT‐PersL in a single material system places even more stringent demands on the energy level structure and defect manipulation of the material. This goal relies on the precisely engineered trap energy level distribution, enabling it to effectively store charge carriers at high temperatures while continuously supplying energy to luminescent centers through controlled energy release.

Therefore, selecting suitable hosts and dopant ions, and precisely controlling the depth and density of traps, is considered a key strategy for achieving high‐efficiency luminescence performance, especially for applications above room temperature. Recently, gallium oxide‐based luminescent materials have attracted substantial interest owing to their excellent chemical robustness and thermal stability [20, 21, 22]. Among these, the spinel‐type LiGa_5_O_8_ has emerged as a prominent host for luminescence. LiGa_5_O_8_ crystallizes in an inverse spinel structure featuring 64 tetrahedral and 32 octahedral voids. In this framework, 8 Ga^3+^ ions occupy the tetrahedral sites, whereas the remaining Ga^3+^ and Li^+^ ions jointly fill half of the octahedral voids, resulting in only 24 cation‐occupied positions. Consequently, a substantial population of intrinsic defects arises, including anti‐site defects [23], oxygen vacancies ($V_{o^{\cdot \cdot }}$) and interstitial oxygen (O_i_″) [24], providing an important structural basis for charge carrier trapping and trap energy level modulation. Simultaneously, the abundance of unfilled voids gives LiGa_5_O_8_ excellent doping flexibility, making it an ideal luminescent matrix. To date, various LiGa_5_O_8_‐based phosphors have been reported to exhibit PersL at room temperature, including LiGa_5_O_8_:Bi^3+^ [25], LiGa_5_O_8_:$Eu^{3^{+}}/Tb^{3^{+}}$ [26], LiGa_5_O_8_:Fe^3+^ [27, 28], LiGa_5_O_8_:Pr^3+^ [29], and LiGa_5_O_8_:Cr^3+^ [30]. Although rare‐earth Tb^3+^‐doped systems have shown improved HT‐PersL [31], its green emission is inherently restricted toTb^3+^’s green f‐f transitions and thus lacks spectral tunability and temperature‐responsive chromatic behavior. On the other hand, while anti‐TQ PL and only a few HT‐PersL have been reported in different material systems, they typically involve different energy modulation processes. Further research is needed to elucidate how efficient charge carrier storage and thermally activated release at high temperatures can be achieved through defect engineering within a single material system, without relying on rare‐earth ions with intrinsically non‐tunable emission, while simultaneously enabling temperature‐dependent multicolor anti‐TQ PL and HT‐PersL.

In this work, a LiGa_5_O_8_ (LGO) phosphor with enhanced high‐temperature luminescence performance is realized through the synergistic co‐doping of Cu^2+^ and Zn^2+^, which reconstructs the intrinsic defect hierarchy of the host lattice. The cooperative incorporation of Cu^2+^ and Zn^2+^ simultaneously modulates the electronic band structure and introduces a high density of deep traps, forming a continuous trap that effectively regulates charge carrier storage and thermally activated release at elevated temperatures. As a result, LGO:0.05%Cu 10% Zn (LGO:Cu,Zn) exhibits pronounced anti‐TQ PL, with the emission intensity at 500 K approximately 9.4 times that at 300 K, together with a significantly enhanced HT‐PersL, whose initial intensity is about 20 times higher than that of LGO at 500 K. In addition, the engineered defect states give rise to a distinct temperature‐dependent color evolution, changing from blue emission at room temperature to red emission at elevated temperatures. This unique combination of defect‐mediated HT PL/PersL and dynamic chromatic response establishes LGO:Cu,Zn as a promising candidate for next‐generation high‐temperature displays, advanced anti‐counterfeiting, and intelligent thermal sensing applications.

## 2 Results and Discussion

### 2.1 Structural Characterization

**FIGURE 1:**
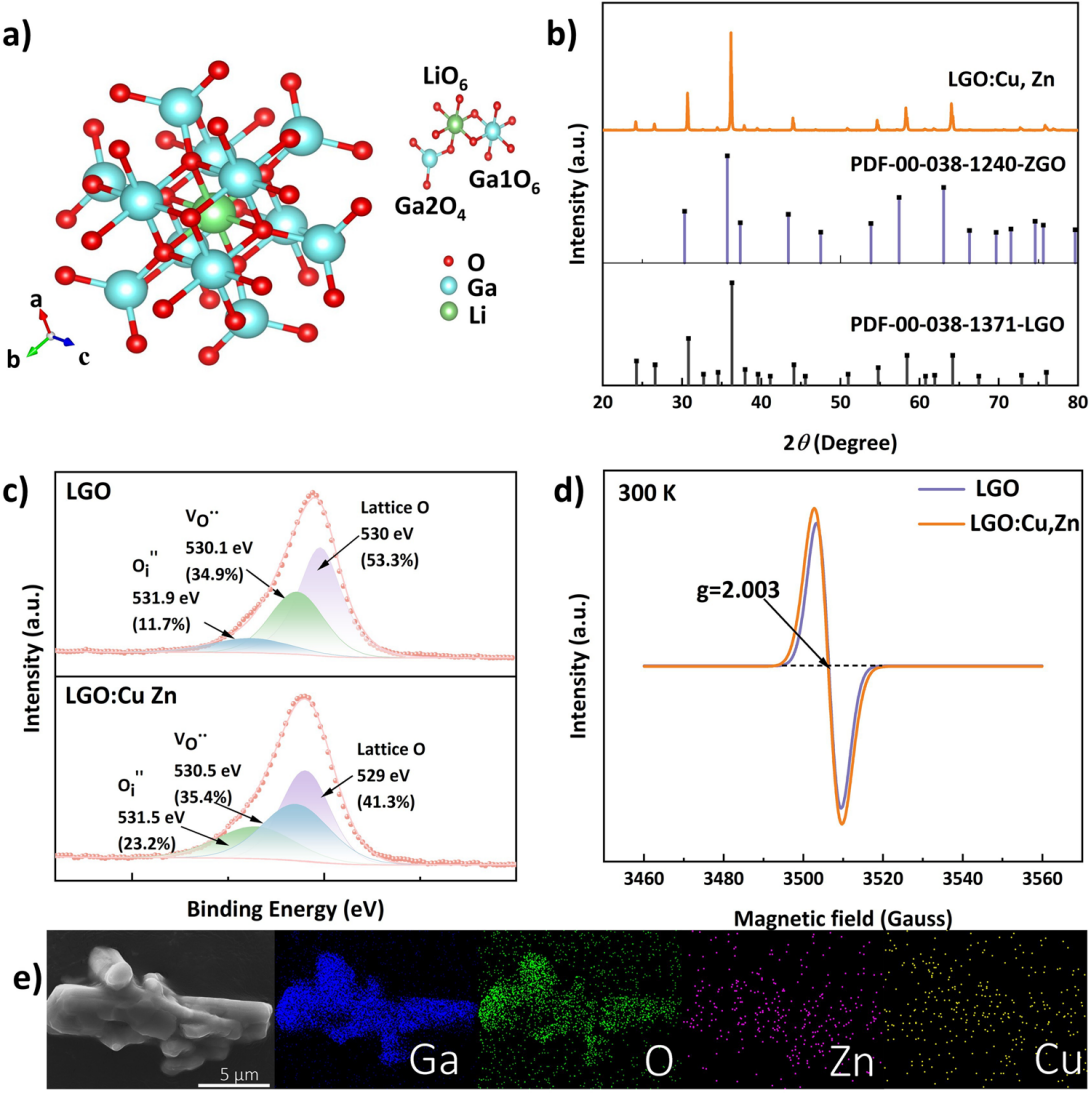
(a) Crystal structure of LGO. (b) XRD patterns of LGO:Cu,Zn. (c) High‐resolution O1s XPS spectra of LGO and LGO:Cu,Zn. (d) Electron paramagnetic resonance (EPR) spectra of LGO and LGO:Cu,Zn. (e) Elemental mapping images of LGO:Cu,Zn.

A series of LiGa_5_O_8_:xCu^2+^ phosphors (x = 0.025%, 0.05%, 0.075%, 0.1%, 0.25% mol) were prepared via high‐temperature solid‐state reactions. Figure 1a shows the crystal structure of LiGa_5_O_8_, an inverse spinel belonging to the P4_3_32 space group. It consists of three fundamental units: [Ga_1_O_6_] octahedra, [LiO_6_] octahedra, and [Ga_2_O_4_] tetrahedra. The XRD patterns for LiGa_5_O_8_:xCu^2+^ are presented in Figure S1a. A magnified view of the diffraction peak near 24° reveals a gradual shift toward higher angles with increasing Cu^2+^ content, indicating lattice contraction. This result is attributed to the substitution of Li^+^ ions (CN = 6, 0.76 Å) by smaller Cu^2+^ ions (CN = 6, 0.73 Å). In contrast, upon Zn^2+^ incorporation, the peak around 30° shifts gradually to lower angles as the Zn^2+^ content increases (Figure S1b), indicating lattice expansion, which demonstrates continuous lattice evolution between the terminal structures [32]. This expansion can be attributed to the substitution of Ga^3+^ ions (CN = 4, 0.47 Å) by larger Zn^2+^ ions (CN = 4, 0.60 Å). The x‐ray diffraction (XRD) pattern of LGO:Cu,Zn (Figure 1b) matches well with the standard patterns of LiGa_5_O_8_ (PDF No. 38–1071) and ZnGa_2_O_4_ (PDF No. 38–1240), confirming the successful synthesis of a phase‐pure solid solution without detectable impurities.

To clarify the effect of Cu^2+^ and Zn^2+^ doping on the crystal structure, Rietveld refinement was performed using LiGa_5_O_8_ as the starting model. The refinement converged well, with reliability factors of R_wp_ = 8.9% and R_p_ = 6.5% for LGO, and R_wp_ = 5.14% and R_p_ = 3.36% for LGO:Cu,Zn, (Figure S2a,b), confirming that all samples adopt the cubic noncentrosymmetric space group P4_3_32 (No. 212). Furthermore, as summarized in Table S1, co‐doping systematically increases the lattice parameters (a, b, c) and cell volume (V) compared to undoped LGO, consistent with the observed XRD peak shifts.

Raman spectroscopy (Figure S3) was employed to examine local structural variations induced by Cu^2+^ and Zn^2+^ doping. The high‐frequency peaks (550‐800 cm^−1^) correspond to Ga─O bonds, while low‐frequency peaks (200–550 cm^−1^) are associated with Li─O vibrations. Although the peak positions remained essentially unchanged for all samples, variations in intensity suggest the presence of local lattice distortion [33, 34]. Specifically, Zn^2+^ substitution for Ga^3+^ leads to a decrease in the intensity of the Ga─O peak, whereas Cu^2+^ substitution at Li^+^ sites results in weakened Li─O vibrations. These observations align well with the XRD results. To investigate the evolution of oxygen defects, x‐ray photoelectron spectroscopy (XPS) was used to analyze O 1s. As shown in Figure 1c, the deconvoluted XPS peaks were assigned to lattice oxygen, $V_{o^{\cdot \cdot }}$, and O_i_″. Both LGO and LGO:Cu,Zn contain $V_{o^{\cdot \cdot }}$ and O_i_″ defects, confirming their coexistence within the lattice. In undoped LGO, the $V_{o^{\cdot \cdot }}$ content in LGO is 34.9%, accompanied by an O_i_″ content of 11.7%. After Cu^2+^ and Zn^2+^ co‐doping, the $V_{o^{\cdot \cdot }}$ content slightly increases to 35.4%, while O_i_″ content increases significantly to 23.2%. EPR measurements (Figure 1d) further confirm the presence of $V_{o^{\cdot \cdot }}$ and the relative concentrations obtained from EPR agree well with the XPS results. The combined XPS and EPR analyses indicate that Cu^2+^ and Zn^2+^ co‐doping mainly increases the concentration of O_i_″.

Furthermore, the corresponding elemental mapping and EDS analysis of LGO:Cu,Zn reveal a uniform distribution of the doped elements, confirming their successful incorporation into the host lattice. Li^+^ is not detected in the EDS spectrum due to electron‐beam ionization effects [35].

### 2.2 PersL Performance

To optimize the Cu^2+^ doping contents, a series of LGO:Cu samples with varying Cu^2+^ concentrations were synthesized. Comparison of their initial PersL intensities after 5 min of 254 nm UV irradiation (Figure S4a) identified 0.05 mol% Cu^2+^ as the optimal concentration. Subsequently, a series of LGO: 0.05%Cu with different Zn^2+^ contents were prepared, and their initial PersL intensities were evaluated (Figure S4b). The decrease in PersL intensity above 10% Zn^2+^ is attributed to a reduction in trap density near 300 K, as evidenced by TL curves (Figure S4c). The maximum intensity was achieved at a Zn^2+^ concentration of 10%. Therefore, a Zn^2+^ concentration of 10%was selected for further investigation.

The optimized LiGa_5_O_8_:0.05%Cu^2+^,10%Zn^2+^ (LGO:Cu,Zn) was then chosen for further study. TL curves of LGO:Cu,Zn were recorded after irradiating the sample under a 254 nm UV irradiation for different durations ranging from 5 to 35 min (Figure S5). The TL intensity increased gradually with irradiation time and reached saturation after 30 min, with no additional enhancement upon prolonged irradiation. Therefore, the optimized 30‐min charging duration was used in all subsequent high‐temperature measurements.

**FIGURE 2:**
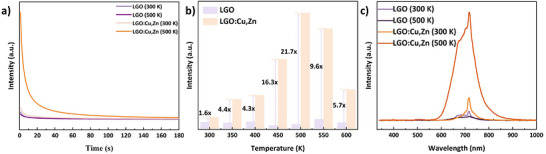
(a) PersL decay curves of LGO and LGO:Cu,Zn measured at 300 and 500 K. (b) Temperature‐dependent initial PersL intensity comparison chart of LGO and LGO:Cu,Zn over the range of 300–600 K (c) PersL emission spectra of LGO and LGO:Cu,Zn recorded at different temperatures (300 and 500 K). (All curves were recorded after 30 min of 254 nm UV irradiation.).

A comparison of their initial PersL intensities during irradiation at 300 and 500 K (Figure 2a) reveals that co‐doping with Cu^2+^ and Zn^2+^ significantly enhances its PersL, especially at 500 K. To further examine the HT‐PersL behavior, temperature‐dependent PersL decay curves were measured over the range of 300–600 K. The decay curves of both LGO and LGO:Cu,Zn phosphors are presented in Figure S6. The LGO shows no obvious temperature‐dependent variation, whereas LGO:Cu,Zn displays a continuous increase in initial PersL intensity with increasing temperature, peaking at 500 K before declining at higher temperatures. As shown in Figure 2b, the initial PersL intensity of LGO:Cu,Zn at 500 K is approximately 20 times that of the undoped LGO. To benchmark the high‐temperature performance, LGO:Cu,Zn was compared with two representative Cr^3+^‐doped persistent phosphors, ZnGa_2_O_4_:Cr (ZGO:Cr) [36] and LiGa_5_O_8_:Cr (LGO:Cr) [21]. As shown in Figure S7, both Cr^3+^‐doped samples exhibit significantly lower HT‐PersL intensity than LGO:Cu,Zn at elevated temperatures, while LGO:Cu,Zn maintains a stronger and more sustained afterglow duration. In addition, LGO:Cr and ZGO:Cr also exhibit thermally enhanced emission behavior upon heating; however, the enhancement is much weaker than that observed for LGO:Cu,Zn (Figure S8). In contrast, LGO:Cu,Zn shows a more pronounced thermal enhancement and stronger HT‐PersL emission at elevated temperatures. The PersL spectra of LGO and LGO:Cu,Zn at two temperatures are shown in Figure 2c; the emission peak position remains unchanged, indicating that the radiative recombination pathway is preserved. However, as the temperature increases, a pronounced broadening of the emission band is observed. This phenomenon is primarily attributed to enhanced exciton‐phonon coupling at elevated temperatures, where increased lattice vibrations lead to fluctuations in the local crystal field around the luminescent centers [37]. As a result, the electronic energy levels involved in radiative recombination become broadened, which manifests as an increase in the full width at half maximum (FWHM) of the emission peak [38].

To further confirm the reliability of the high‐temperature persistent luminescence, cyclic tests were performed at 500 K after the above characterizations. As shown in Figure S9, the sample was charged for 30 min with 254 nm UV before each measurement, and its afterglow decay curves were recorded during five consecutive heating‐cooling cycles. The initial PersL intensity and decay kinetics remained almost unchanged across all cycles, demonstrating excellent repeatability and thermal stability of LGO:Cu,Zn at elevated temperatures.

### 2.3 PL Performance

**FIGURE 3:**
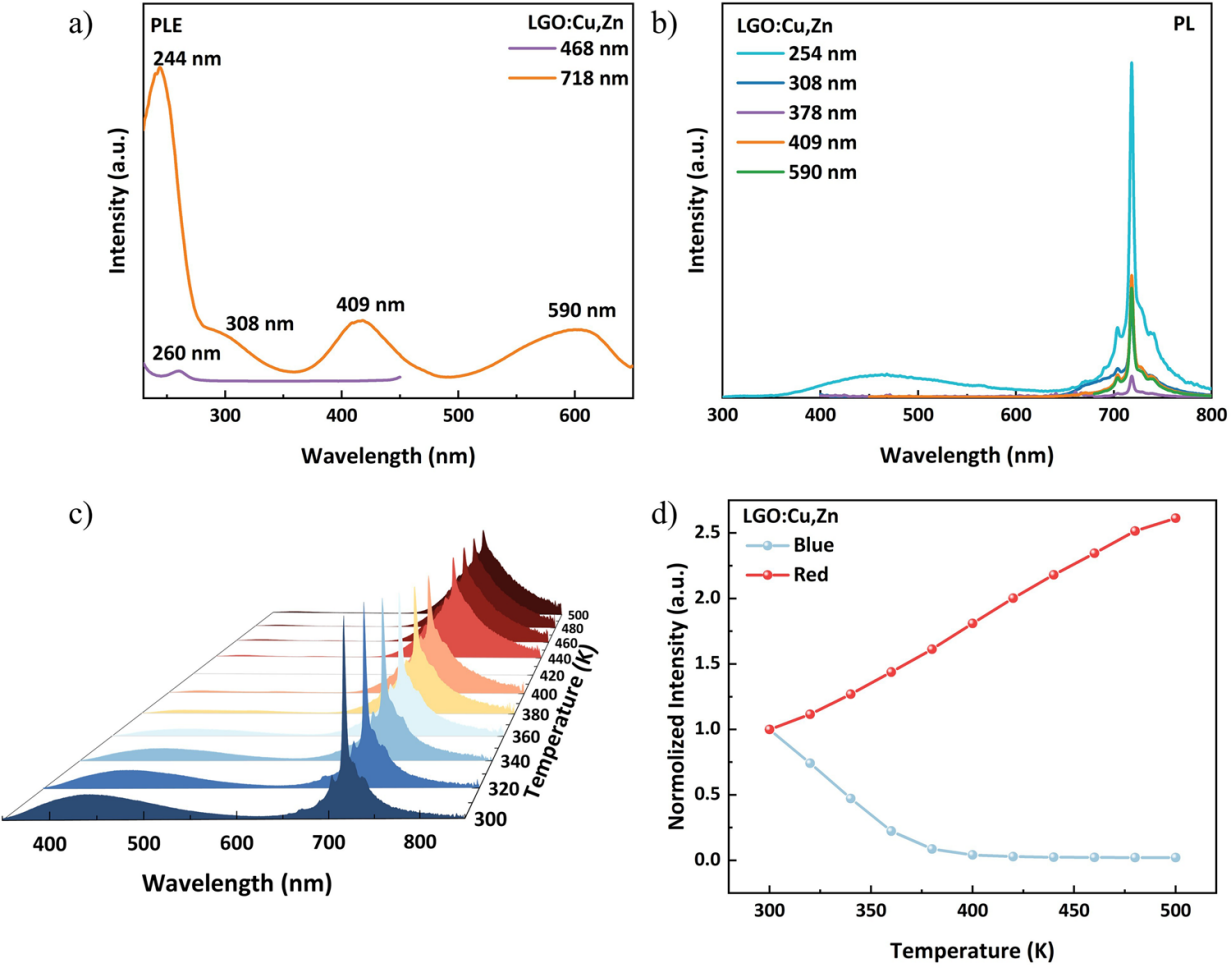
(a, b) PLE and PL spectra of LGO:Cu,Zn. (c) Temperature‐dependent PL spectra of LGO:Cu,Zn recorded under 254 nm excitation from 300 to 500 K. (d) Normalized integrated intensities of the blue and red emission bands as a function of temperature.

To further study the luminescence behavior of LGO:Cu,Zn, PL and PL excitation (PLE) spectra were recorded, as shown in Figure 3a,b, respectively. The PL spectrum consists of a broad emission band at 400–600 nm and a distinct red emission centered at 718 nm, which are attributed to [GaO_4_] and O_i_″^,^ respectively [23, 24]. When monitoring the red emission at 718 nm, the corresponding PLE spectra exhibit four peaks at 244, 308, 409, and 590 nm, which can be assigned to host lattice absorption, $V_{o^{\cdot \cdot }}$, [GaO_4_] tetrahedron transition, and $V_{o^{\cdot \cdot }}$ defect energy level transition, respectively [24]. By comparing the PL spectra of LGO and LGO:Cu,Zn under 254 nm excitation (Figure S10), it can be observed that the emission peak positions remain unchanged after co‐doping. Furthermore, the PersL spectra of LGO:Cu,Zn (Figure 2c) show a dominant emission peak located at 718 nm, consistent with the PL emission. These results indicate that Cu^2+^ and Zn^2+^ do not act as luminescence centers but instead modify the defect‐related emission processes.

To assess the high‐temperature PL performance of LGO:Cu,Zn, temperature‐dependent PL spectra were recorded from 300 to 500 K. As shown in Figure 3c, the blue emission displays typical TQ, while the red emission shows anti‐TQ, with its intensity gradually increasing as the temperature rises. Figure 3d summarizes the integrated intensities of blue (300–600 nm) and red (600–800 nm) at each temperature. With increasing temperature, the blue emission decreases monotonically, whereas the red emission increases continuously, leading to an initial slight decrease followed by an overall enhancement of the total emission intensity (Figure S11). Such a temperature‐dependent enhancement of the red emission is indicative of a pronounced anti‐TQ PL behavior. To verify the reversibility and thermal stability of the anti‐TQ behavior, three consecutive heating‐cooling cycles were performed by tracking the integrated red emission during temperature variation (Figure S12). Similar thermal enhancement behavior was observed during all three cycles, indicating good reversibility and thermal stability of the anti‐TQ process. In addition, variable‐temperature XRD measurements performed between 300 and 500 K (Figure S13) show no detectable phase transition, further confirming the robust structural stability of LGO:Cu,Zn.

To clarify whether this unusual PL behavior originates from the Cu^2+^/Zn^2+^ co‐doping. PL spectra (Figure S14) were recorded at 300, 400, and 500K. At all measured temperatures, LGO also exhibits thermal enhancement of emission intensity at elevated temperatures. In contrast, LGO:Cu,Zn shows a much stronger red emission, which increases progressively with temperature and is consistently higher than that of LGO. By contrast, after annealing LGO:Cu,Zn under an Ar atmosphere, the PL intensity is strongly suppressed over the entire spectral range, indicating that the temperature‐enhanced red emission is closely associated with oxygen‐related defect states introduced by Cu^2+^/Zn^2+^ co‐doping. These results confirm that Cu^2+^ and Zn^2+^ co‐doping simultaneously enhances both PL and PersL. The internal quantum efficiency (IQE), absorption efficiency (AE), and external quantum efficiency (EQE) of LGO:Cu,Zn were measured using an integrating sphere method [39, 40] to be 25.65%, 40.60%, and 10.41%, respectively, confirming the efficient luminescence of the co‐doped sample.

### 2.4 Luminescence Mechanism

**FIGURE 4:**
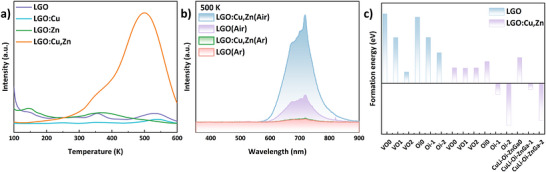
(a) TL spectra of the LGO, LGO:Cu, LGO:Zn, and LGO:Cu,Zn. (b) PersL emission spectra of LGO and LGO:Cu,Zn measured at 500 K. (c) Calculated formation energies of oxygen‐related defects in LGO and LGO:Cu,Zn.

To elucidate the HT‐PersL mechanism of LGO:Cu,Zn, TL curves were systematically recorded for LGO, single‐doped samples (LGO:Cu and LGO:Zn), and LGO:Cu,Zn, as shown in Figure 4a. LGO exhibits a TL peak near 500 K, indicating the presence of deep traps; however, their density is relatively low. Upon Cu^2+^ doping alone, a TL peak appears between 500–600 K, suggesting the formation of deep traps, albeit with lower intensity than that of undoped LGO. In contrast, Zn^2+^‐only doping shifts the TL peak to the 300–400 K range, indicating a shallower trap depth. Remarkably, only Cu^2+^ and Zn^2+^ co‐doping enables simultaneous regulation of both trap depth and trap density, leading to deeper and more abundant trap states capable of storing a larger population of charge carriers, which are gradually released upon heating, thereby enhancing HT‐PersL. As discussed earlier, the concentration of O_i_″ increased significantly after co‐doping (Figure 1c). In order to further validate the regulatory role of O_i_″, the content of O_i_″ was reduced by altering the sintering atmosphere. Samples were sintered under Ar gas at the same temperatures, and their TL and PersL behaviors were compared (Figure S15). XPS fitting of the Ar‐sintered samples reveals a substantial increase in $V_{o^{\cdot \cdot }}$ content alongside a significant reduction in O_i_″ content (Figure S16). Correspondingly, both blue and red light emission intensities decrease markedly. Although the blue emission is often attributed to $V_{o^{\cdot \cdot }}$ in previous reports [24], in the present system it originates from [GaO_4_]. The weakened blue emission under Ar atmosphere can be attributed to $V_{o^{\cdot \cdot }}$ formed, which disrupts the [GaO_4_] structure. Similarly, the pronounced attenuation of 718 nm red emission intensity under Ar further confirms its origin from O_i_″.

Figure 4b compares the PersL spectra of LGO and LGO:Cu,Zn at 500 K under air and Ar atmospheres. It can be clearly observed that the intensity of LGO:Cu,Zn sintered in air is significantly higher than that of other materials. This enhancement is attributed to the combined effects of air sintering and Cu^2+^/Zn^2+^ co‐doping, which promote the formation of O_i_″ while preserving deep trap states. The improved HT‐ PersL stems from increased deep traps, which store charge carriers efficiently and release them progressively with increasing temperature.

The deterioration of luminescence performance of co‐doped samples after Ar sintering indicates that O_i_″ not only acts as a luminescence center but is also indispensable for the formation of deep traps. Based on these observations, we propose that defect clusters, possibly of the type Zn_Ga_^'^‐O_i_″‐Cu_Li_^·^, play a dominant role in the HT‐PersL and anti‐TQ behavior by serving as the main deep‐level trapping centers. These defect clusters enable efficient charge carrier storage and gradual thermal release at elevated temperatures.

Using Shalgaonkar's approximation (*E*  = *T_m_* /500 [10, 41]), where T_m_ is the TL peak temperature, the trap depth at 500 K is estimated to be ∼1 eV. Charge carriers trapped at such deep levels encounter significantly higher escape barriers than those in shallow traps at room temperature, accounting for the enhanced HT‐PersL.

The optical absorption properties of LGO and LGO:Cu,Zn, were further examined by the ultraviolet‐visible diffuse reflectance spectra (UV‐DRS) (Figure S17). Bandgap were determined using the Tauc plot method [42] by extrapolating the linear region of (αhν)^2^ versus hν. The obtained direct bandgap are 5.3 eV for LGO, 4.59 eV for LGO:Cu,Zn, indicating that co‐doping significantly narrows the bandgap. Such bandgap narrowing facilitates carrier excitation and trapping under UV irradiation, allowing a larger population of trapped electrons to participate in subsequent thermally stimulated recombination processes, which is beneficial for the enhancement of PersL.

To further investigate the electronic structure of LGO and LGO:Cu,Zn, Density functional theory (DFT) calculations were carried out. The projected density of states (PDOS) (Figure S18) results show that the valence band maximum (VBM) of LGO is mainly derived from O‐2p orbitals, while the conduction band minimum (CBM) is composed of O‐2p and Ga‐4p orbitals. In LGO:Cu,Zn, the VBM remains primarily derived from O‐2p orbitals, whereas the CBM arises from the combined contributions of O‐2p, Ga‐4p, Zn‐3d, and Cu‐3d orbitals. Correspondingly, the calculated band structure of LGO:Cu,Zn reveals an upward shift of the VBM, resulting in a reduced band gap. Although the absolute band gap values obtained using the PBE functional deviate from the experimental values, the calculated trend agrees well with the experimentally observed band gap narrowing upon co‐doping (Figure S19).

The effect of Cu^2+^ and Zn^2+^ co‐doping on defect stability was further evaluated by comparing oxygen defect formation energies before and after doping (Figure 4c). Compared with LGO, Cu^2+^ and Zn^2+^ co‐doping significantly lowers the formation energy of O_i_″, and the resulting defect clusters Zn_Ga_^'^‐O_i_″‐Cu_Li_^·^ are energetically stable. The selection of Cu^2+^ and Zn^2+^ as co‐dopants is closely related to their complementary roles in defect regulation within the inverse spinel LGO lattice. Zn^2+^ preferentially substitutes for Ga^3+^, introducing negatively charged defects, whereas Cu^2+^ tends to occupy Li^+^ sites and introduces positively charged defect centers. The electrostatic interaction between these oppositely charged defects favors the formation of defect clusters rather than isolated point defects. Such defect association stabilizes oxygen‐related defects and promotes the development of a higher density of deep traps. This cooperative defect reconstruction is difficult to achieve through single‐element doping or isovalent substitution alone, and provides a plausible origin for the simultaneous enhancement of anti‐TQ PL and HT‐PersL observed in LGO:Cu,Zn.

**FIGURE 5:**
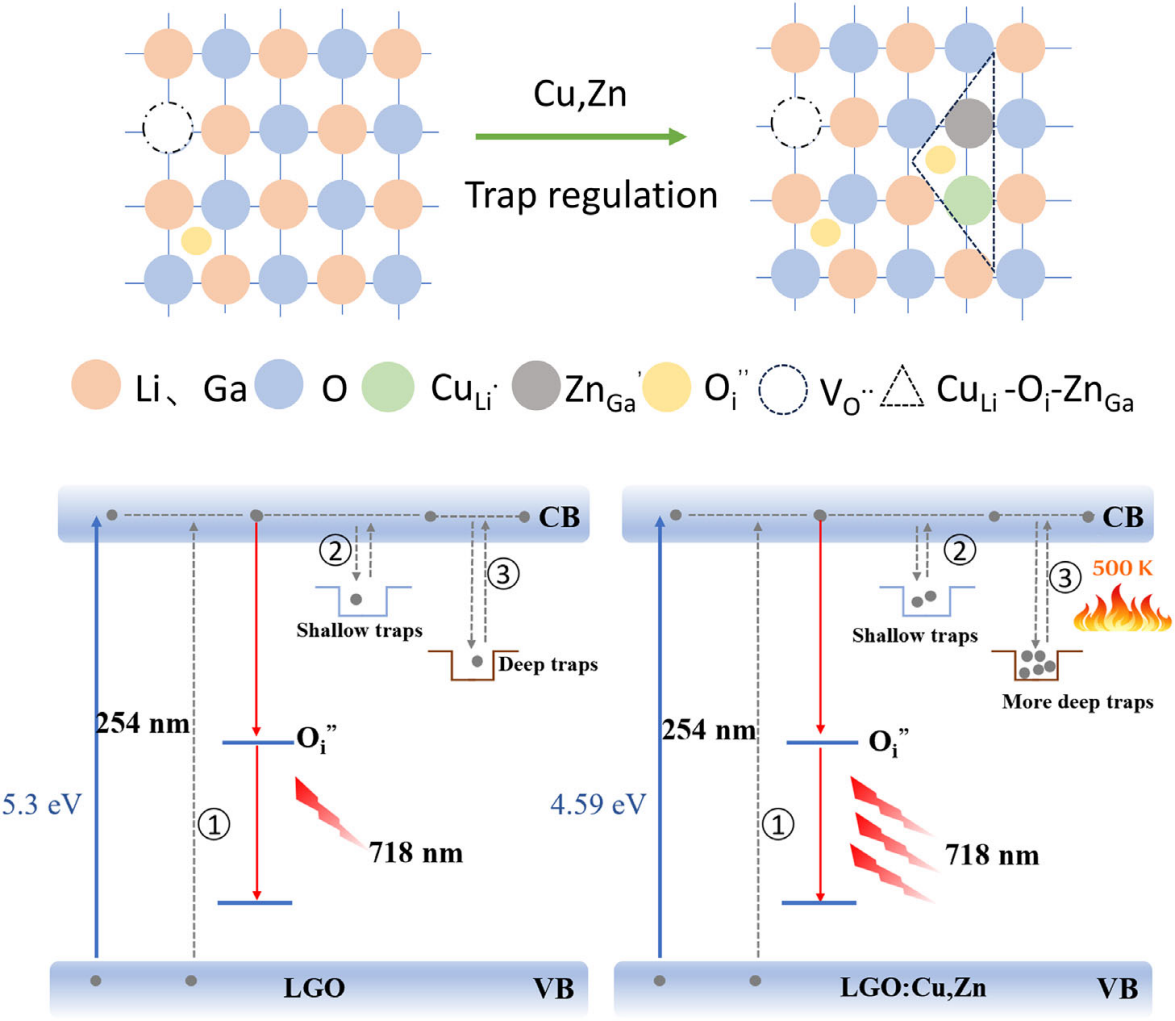
Schematic illustration of the defect‐regulated luminescence mechanism.

To further elucidate the mechanism of PersL, schematic diagrams are used to illustrate the structural evolution before and after co‐doping, accompanied by a notable increase in deep trap density (Figure 4a). Based on comprehensive experimental results discussed above, a possible mechanism is depicted in Figure 5. Under 254 nm ultraviolet irradiation, electrons are excited from the VB to the CB (process 1). A fraction of the excited electrons is subsequently captured by shallow and deep traps (process 2). As the temperature rises, trapped electrons are thermally released and recombine radiatively at O_i_″, giving rise to sustained PersL (process 3).

## 3 Application

**FIGURE 6:**
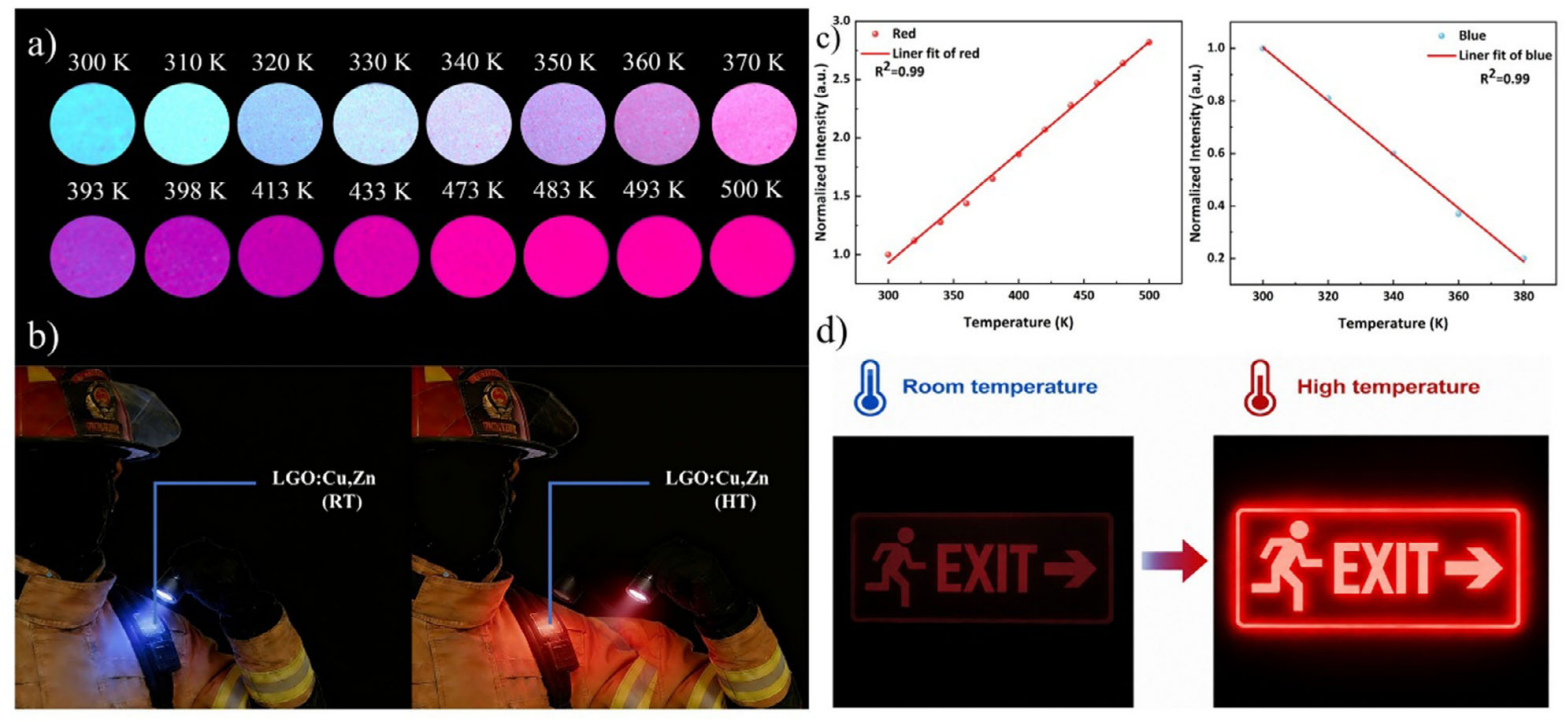
(a) PL colours at different temperatures (b) Schematic diagram for firefighting applications (c) Red light fitting curve and blue light fitting curve (d) Schematic diagram of the high‐temperature emergency escape indicator.

Leveraging the thermal stability and temperature‐dependent luminescence of LGO:Cu,Zn, its potential application in temperature monitoring and anti‐counterfeiting was explored. Luminescence temperature indicators are of particular interest for safety‐critical scenarios. In firefighting operations, the outer layer of thermal protective clothing (the fire‐facing side) can be exposed to transient temperatures of several hundred degrees Celsius, whereas the inner layer (the body‐facing side) must remain within a safe temperature range. Human skin tolerance is approximately 317 K. Although temperatures slightly above this threshold may not immediately cause burns, it indicates severe degradation of the thermal insulation performance and represents a significant safety risk. Therefore, a temperature indicator exploiting the thermochromic luminescence behavior of LGO:Cu,Zn was developed. At room temperature, it exhibits blue emission. Upon heating, the blue emission fades, while red emission is thermally enhanced. As demonstrated in Figure 6a, heating initially produces mixed emission, with a complete transition to red at 370 K.

As illustrated in Figure 6b, the reversible color‐changing materials (patches or coatings) are embedded within the lining or interlayer at critical garment zones (shoulders, chest, back, knees). A transition temperature of 370 K is selected, as it lies well above the safe comfort range while remaining below the threshold for irreversible material damage. After firefighting operations, firefighters or equipment managers can rapidly scan the garment lining using a portable ultraviolet lamp in a secure rear area. The appearance of red emission under excitation at specific locations indicates the inner layer temperature has reached or exceeded the 370 K warning threshold, prompting further inspection or replacement of the equipment. For potential temperature‐sensing applications, linear fittings of the blue and red emission intensities as a function of temperature show good linearity over the investigated range (Figure 6c), suggesting that LGO:Cu,Zn is a promising candidate for optical temperature sensing. To demonstrate the potential application of HT‐PersL, a passive emergency indication concept was designed based on the temperature‐enhanced red persistent luminescence of LGO:Cu,Zn (Figure 6d). Under elevated temperatures, the material exhibits significantly enhanced red afterglow intensity, enabling clear optical warning and emergency indication even after removal of the excitation source. Such behavior highlights its potential for safety indication in thermally harsh environments.

## 4 Conclusion

In summary, an effective defect‐engineering strategy has been demonstrated to achieve anti‐TQ PL and HT‐PersL simultaneously in a single LGO system. Synergistic of Cu^2+^/Zn^2+^ co‐doping reconstructed deep traps capable of efficient charge carrier storage and thermally activated release at elevated temperatures, leading to improved luminescence stability. Furthermore, trap regulation induces a temperature‐dependent color evolution from blue to red emission. These results indicate that rational control of defect types and their interactions provides an effective route to simultaneously regulating PL and PersL at high temperatures. This work provides a practical approach for the development of luminescent materials designed for thermally harsh environments.

## Author Contributions

**Yuchen Wang**: Writing – original draft, conceptualization, investigation, methodology, formal analysis, data curation, validation, visualization. **Yilong Fan**: visualization, validation, methodology, investigation, formal analysis. **Jingui Duan**: supervision, funding acquisition. **Tengxiang Long**: investigation, formal analysis. **Dingling Xiong**: investigation, formal analysis. **Lina Su**: formal analysis, resources. **Ling Huang**: supervision, writing – review and editing, funding acquisition. **Jin Zhang**: investigation, formal analysis. **Hao Song**: investigation, formal analysis. **Jianhua Liu**: writing – review and editing, funding acquisition, project administration, supervision, resources, writing – original draft. **Pengpeng Dai**: resources. **Chao Sun**: investigation, formal analysis.

## Conflicts of Interest

The authors declare no conflicts of interest.

## Supporting information




**Supporting File**: advs76549‐sup‐0001‐SuppMat.docx.

## Data Availability

The data that support the findings of this study are available on request from the corresponding author. The data are not publicly available due to privacy or ethical restrictions.

## References

[1] Y. Zhuang , Y. Lv , L. Wang , et al., “Trap Depth Engineering of SrSi_2_O_2_N_2_:Ln^2+^,Ln^3+^ (Ln^2+^ = Yb, Eu; Ln^3+^ = Dy, Ho, Er) Persistent Luminescence Materials for Information Storage Applications,” ACS Applied Materials & Interfaces 10 (2018): 1854–1864, 10.1021/acsami.7b17271. 29277986

[2] T. Seto , Y. Wang , J. Wu , and Z. Li , “Progress of M_2_Si_5_N_8_:Eu Series in Industrial LED Phosphors,” Journal of Materials Chemistry C 11 (2023): 6512–6527, 10.1039/D2TC05284A.

[3] Y. Yang , W. Gao , M. Zhu , et al., “Thermally Boosted Luminescent From a Novel Red Phosphor With Two‐Dimensional Negative Thermal Expansion for High‐Quality WLEDs,” Journal of Alloys and Compounds 1045 (2025): 184638, 10.1016/j.jallcom.2025.184638.

[4] C. R. Y. Zhao , F. Pietra , K. Rolf , M. Celso , and M. Andries , “High‐Temperature‐Luminescence‐Quenching‐of‐Colloidal‐Quantum‐Dots,” ACS Nano 6 (1983): 9058–9067. 10.1021/nn303217q 22978378

[5] M. Lax , “The Franck‐Condon Principle and Its Application to Crystals,” The Journal of Chemical Physics 20 (1952): 1752–1760, 10.1063/1.1700283.

[6] J. M. F. van Dijk and M. F. H. Schuurmans , “On the Nonradiative and Radiative Decay Rates and a Modified Exponential Energy Gap Law for 4f–4f Transitions in Rare‐Earth Ions,” The Journal of Chemical Physics 78 (1983): 5317.

[7] A. Abdukayum , J. T. Chen , Q. Zhao , and X. P. Yan , “Functional Near Infrared‐Emitting Cr^3+^/Pr^3+^ Co‐Doped Zinc Gallogermanate Persistent Luminescent Nanoparticles With Superlong Afterglow for In Vivo Targeted Bioimaging,” Journal of the American Chemical Society 135 (2013): 14125–14133, 10.1021/ja404243v. 23988232

[8] Z. Z. Chen , L. C. Wang , D. Manoharan , et al., “Low Dose of X‐Ray‐Excited Long‐Lasting Luminescent Concave Nanocubes in Highly Passive Targeting Deep‐Seated Hepatic Tumors,” Advanced Materials 31 (2019): 1905087, 10.1002/adma.201905087. 31625638

[9] S. Hirata , K. Totani , J. Zhang , et al., “Efficient Persistent Room Temperature Phosphorescence in Organic Amorphous Materials Under Ambient Conditions,” Advanced Functional Materials 23 (2013): 3386–3397, 10.1002/adfm.201203706.

[10] F. Peng , T. Seto , and Y. Wang , “First Evidence of Electron Trapped Ln^2+^ Promoting Afterglow on Eu^2+^, Ln^3+^ Activated Persistent Phosphor‐Example of BaZrSi_3_O_9_:Eu^2+^, Sm^3+^ ,” Advanced Functional Materials 33 (2023): 2300721, 10.1002/adfm.202300721.

[11] R. E. Rojas Hernandez , F. Rubio Marcos , M. Á. Rodriguez , and J. F. Fernandez , “Long Lasting Phosphors: SrAl_2_O_4_:Eu, Dy as the Most Studied Material,” Renewable and Sustainable Energy Reviews 81 (2018): 2759–2770, 10.1016/j.rser.2017.06.081.

[12] Y. Wang , P. Dang , L. Qiu , et al., “Multimode Luminescence Tailoring and Improvement of Cs_2_NaHoCl_6_ Cryolite Crystals Via Sb^3+^/Yb^3+^ Alloying for Versatile Photoelectric Applications,” Angewandte Chemie International Edition 62 (2023): 202311699, 10.1002/anie.202311699. 37724623

[13] X. Zhou , K. Han , Y. Wang , et al., “Energy‐Trapping Management in X‐Ray Storage Phosphors for Flexible 3D Imaging,” Advanced Materials 35 (2023): 2212022, 10.1002/adma.202212022. 36807928

[14] A. Akbar , J. Zeler , A. Owczarek , and J. Brgoch , “Enabling High‐Temperature Persistence Luminescence in Ca_2_Si_5_N_8_:Eu^2+^, RE^3+^ via Trap State Engineering,” Advanced Optical Materials 13 (2025): 02228, 10.1002/adom.202502228.

[15] Z. Liu , L. Zhao , X. Yang , et al., “Long Persistent Luminescence Properties of NaBaScSi_2_O_7_: Tb^3+^ and it's Applications Above Room Temperature,” Chemical Engineering Journal 401 (2020): 126119, 10.1016/j.cej.2020.126119.

[16] Y. Zhuang , J. Ueda , and S. Tanabe , “Tunable trap depth in Zn(Ga_1−x_Alx)_2_O_4_:Cr,Bi Red Persistent Phosphors: Considerations of High‐Temperature Persistent Luminescence and Photostimulated Persistent Luminescence,” Journal of Materials Chemistry C 1 (2013): 7849, 10.1039/c3tc31462f.

[17] Y. Zhou , Y. Li , H. Wu , X. Li , and M. Gou , “High Temperature Persistent Luminescence in Tb^3+^ Doped CaSr_2_Al_2_O_6_ Phosphor,” Optik 242 (2021): 167103, 10.1016/j.ijleo.2021.167103.

[18] Z. Long , J. Zhou , J. Qiu , et al., “Thermally Stable Photoluminescence and Long Persistent Luminescence of Ca_3_Ga_4_O_9_:Tb^3+^/Zn^2+^ ,” Journal of Rare Earths 36 (2018): 675–679, 10.1016/j.jre.2017.11.016.

[19] X. Yu , S. Wang , Y. Zhu , et al., “High‐Temperature Long Persistent and Photo‐Stimulated Luminescence in Tb^3+^ Doped Gallate Phosphor,” Journal of Alloys and Compounds 701 (2017): 774–779, 10.1016/j.jallcom.2017.01.210.

[20] Y. Zhang , Z. Han , Y. Guan , et al., “Rare Earth Element Applications in Ga_2_O_3_: Luminescence and Scintillation,” Applied Physics Reviews 12 (2025): 021324, 10.1063/5.0258406.

[21] G. Pan , Y. Wang , J. Wang , et al., “Tunable Broadband Near‐Infrared Luminescence From Cr^3+^‐Doped Gallium Oxide‐Based Phosphors for Advanced Sensing and LED Applications,” Inorganic Chemistry Frontiers 11 (2024): 3941–3949, 10.1039/D4QI00846D.

[22] Q. Guo , K. Saito , and T. Tanaka , “Color‐Tunable Light‐Emitting Diodes Based on Rare Earth Doped Gallium Oxide Films,” ACS Applied Electronic Materials 5 (2023): 4002–4013, 10.1021/acsaelm.3c00587.

[23] N. Zhang , B. Tian , Z. Wang , et al., “Intense Mechanoluminescence in Undoped LiGa_5_O_8_ With Persistent and Recoverable Behaviors,” Advanced Optical Materials 9 (2021): 2100137, 10.1002/adom.202100137.

[24] M. Jia , X. Zhang , X. Yang , et al., “The Self‐Activated LiGa_5_O_8_ Storage Phosphor: Insights Into Its Photo/Thermo/Mechano‐Stimulated NIR Luminescence,” Journal of Materials Chemistry C 13 (2025): 4616–4625, 10.1039/D4TC04818K.

[25] Z. Yi , P. Liu , X. Liu , and Y. Xu , “Prolonged Red Persistent Luminescence in Bi^3+^ Single‐Doped LiGa_5_O_8_: Regulating Traps by Site Selective Occupation,” Inorganic Chemistry 62 (2023): 19542–19551, 10.1021/acs.inorgchem.3c02720. 37971901

[26] P. Zhang , W. Xie , Z. Wang , et al., “Time‐Dependent Dynamic Multicolor Afterglow of Simple LiGa_5_O_8_:Eu^3+^/Tb^3+^Particles for Advanced Anticounterfeiting and Encryption,” Inorganic Chemistry Frontiers 9 (2022): 4022–4029, 10.1039/D2QI00836J.

[27] X. Lu , Y. Wang , Z. Guo , and W. Wang , “Influence of Co‐Doped Si^4+^ on the Near‐Infrared Persistent LiGa_5_O_8_: Fe^3+^ ,” Ceramics International 51 (2025): 31861–31869, 10.1016/j.ceramint.2025.04.378.

[28] X. Lu , Y. Wang , J. Yang , P. D. Townsend , and D. Hreniak , “LiGa_5_O_8_: Fe^3+^: A Novel and Super Long Near‐Infrared Persistent Material,” Ceramics International 50 (2024): 35359–35367, 10.1016/j.ceramint.2024.06.345.

[29] P. Xiong , M. Peng , K. Qin , F. Xu , and X. Xu , “Visible to Near‐Infrared Persistent Luminescence and Mechanoluminescence From Pr^3+^ ‐Doped LiGa_5_O_8_ for Energy Storage and Bioimaging,” Advanced Optical Materials 7 (2019): 1901107, 10.1002/adom.201901107.

[30] Y. J. Chuang , Z. Zhen , F. Zhang , et al., “Photostimulable Near‐Infrared Persistent Luminescent Nanoprobes for Ultrasensitive and Longitudinal Deep‐Tissue Bio‐Imaging,” Theranostics 4 (2014): 1112–1122, 10.7150/thno.9710. 25285164 PMC4173761

[31] P. Zhang , X. Chen , Y. Bai , et al., “Quasi‐Continuous Defect Levels in Broadband Gap: A New Strategy for High‐Temperature Long Persistent Luminescence Materials,” Advanced Optical Materials 12 (2023): 2301406, 10.1002/adom.202301406.

[32] S. Lyu , P. Zhou , J. Du , et al., “Manipulating Trap Distribution and Density by Chemical Unit Cosubstitution for Near‐Infrared Persistent Luminescent Zn_1−2x_Li_x_Ga_2+x_O_4_:Cr^3+^ Solid Solutions,” Journal of Materials Chemistry C 10 (2022): 18404–18414, 10.1039/D2TC03741F.

[33] X. Lu , W. Bian , Y. Li , H. Zhu , Z. Fu , and Q. Zhang , “Influence of Inverse Spinel Structured CuGa_2_O_4_ on Microwave Dielectric Properties of Normal Spinel ZnGa_2_O_4_ Ceramics,” Journal of the American Ceramic Society 101 (2017): 1646–1654, 10.1111/jace.15264.

[34] P. Yadav , S. Uniyal , S. Uma , and R. Nagarajan , “Ordered LiGa_5_O_8_ Loaded With Redox Capable Cu^2+^, Cr^3+^ Ions to Manifest Interesting Optical, Magnetic, and Catalytic Properties,” Journal of Materials Science 56 (2021): 20111–20125, 10.1007/s10853-021-06572-z.

[35] C. Yuan , R. Li , Y. Liu , et al., “Efficient and Broadband LiGaP_2_O_7_:Cr^3+^ Phosphors for Smart Near‐Infrared Light‐Emitting Diodes,” Laser & Photonics Reviews 15 (2021): 2100227, 10.1002/lpor.202100227.

[36] S. K. Sharma , A. Bessière , N. Basavaraju , et al., “Interplay Between Chromium Content and Lattice Disorder on Persistent Luminescence of ZnGa_2_O_4_:Cr^3+^ for In Vivo Imaging,” Journal of Luminescence 155 (2014): 251–256, 10.1016/j.jlumin.2014.06.056.

[37] J. Bhattacharjee and S. D. Singh , “Temperature Dependence of Red Luminescence in Pure β‐Ga_2_O_3_: An Estimation of Electron‐Phonon Interaction,” Solid State Communications 352 (2022): 114831, 10.1016/j.ssc.2022.114831.

[38] L. Tang , Y. Zhang , C. Liao , et al., “Temperature‐Dependent Photoluminescence of CdS/ZnS Core/Shell Quantum Dots for Temperature Sensors,” Sensors 22 (2022): 8993, 10.3390/s22228993. 36433589 PMC9698013

[39] M. Abraham , J. Dhanuka , S. Som , M. K. Pandey , and S. Das , “A Highly Efficient Deep Red‐Emitting Mn^4+^‐Powered Oxyfluoride Nanophosphor Developed for Plant Growth and Optical Thermometric Applications,” Nanoscale 16 (2024): 10690–10705, 10.1039/D4NR00787E. 38695807

[40] M. Abraham , K. K. Thejas , A. K. Kunti , et al., “Strategically Developed Strong Red‐Emitting Oxyfluoride Nanophosphors for Next‐Generation Lighting Applications,” Advanced Optical Materials 12 (2024): 2302580, 10.1002/adom.202401356.

[41] N. Chandrasekhar , K. B. Singh , and R. K. Gartia , “On the Urbach's Formula for Evaluation of Electron Trapping Parameter: The Case of Persistent Luminescent Materials,” Journal of Rare Earths 35 (2017): 733–738, 10.1016/S1002-0721(17)60970-0.

[42] L. Liu , S. Peng , Y. Guo , et al., “Designing X‐Ray‐Excited UVC Persistent Luminescent Material via Band Gap Engineering and Its Application to Anti‐Counterfeiting and Information Encryption,” ACS Applied Materials & Interfaces 14 (2022): 41215–41224, 10.1021/acsami.2c12407. 36064349

