# Supplementary material for: High‐Temperature Persistent Luminescence and Anti‐Thermal Quenching in LiGa5O8 by Trap Engineering
Source: Adv Sci (Weinh). 2026 Jul 11;13(55):e76549. doi: 10.1002/advs.76549 (PMC13355925; doi:10.1002/advs.76549)
Supplement: Supplementary file 1 [file ADVS-13-e76549-s001.docx]

**Supporting Information**

**High-Temperature Persistent Luminescence and Anti-Thermal Quenching in LiGa_5_O_8_:Cu,Zn by Trap Engineering**

Yuchen Wang^†^, Yilong Fan^†^, Jianhua Liu*, Jin Zhang, Dingling Xiong, Tengxiang Long, Lina Su, Chao Sun, Hao Song, Pengpeng Dai, Jingui Duan*, Ling Huang*

(^†^These authors contribute equally to this work)

Y. Wang, Y. Fan, J. Liu, J. Zhang, D. Xiong, T. Long, L. Su, J. Duan, L. Huang

State Key Laboratory of Chemistry and Utilization of Carbon-Based Energy Resources, College of Chemistry, Xinjiang University, Urumqi 830017, China

E-mail: jianhua.liu@xju.edu.cn, duanjingui@xju.edu.cn, lhuang@xju.edu.cn

H. Song, C. Sun, L. Huang

School of Chemistry and Molecular Engineering, Nanjing Tech University, Nanjing 211816, China

P. Dai

Department of Physics and Electronic Engineering & School of Chemistry and Chemical Engineering, Key Laboratory for Mineral Luminescence and Microstructure of Autonomous Region, Xinjiang Normal University, 102 New Hospital Street, Urumqi 830054, China

**Experimental Section**

*Materials preparation:* A series of phosphors (LiGa_5_O_8_: xCu, x = 0.025%, 0.05%, 0.075%, 0.1%, 0.25% mol) were prepared via high-temperature solid-state reactions. The raw materials Li_2_CO_3_ (Aladdin AR), Ga_2_O_3_ (Aladdin 99.99%)_,_ and CuO (Aladdin 99.9%) were weighed according to the stoichiometric ratio and mixed with anhydrous ethanol in an agate mortar. The mixed powder was then transferred to an alumina crucible and heated to 1200°C at a rate of 3 °C/min for 4 h.

LiGa_5_O_8_:0.05%Cu,yZn (y=10%-50%) phosphors were synthesized via high-temperature solid-state reactions. Stoichiometric quantities of Li_2_CO_3_, Ga_2_O_3_, ZnO (Aladdin 99.99%), and CuO were precisely weighed, combined with anhydrous ethanol, and homogenized by grinding in an agate mortar. The mixed precursor was loaded into alumina crucibles and fired at 1200 °C for 4 h in air (heating rate: 3 °C/min).

*Structure and morphology characterization:* Crystallographic phases of the obtained samples were recorded on a Smart Lab SE X-ray powder diffractometer (XRD) using Cu Ka1 radiation at room temperature. The XRD data were collected with the 2θ range from 10° to 80°. The morphology and elemental distribution of the solid solutions were characterized using a scanning electron microscope (SEM) (S-4800, Hitachi, Japan). The XPS measurements were performed using an X-ray photoelectron spectrometer (Thermo Scientific K-Alpha). The PL, PLE spectra, and decay curves were measured at room temperature using. Raman measurements were performed via a HORIBA HR Evolution spectrometer (France), utilizing a 532 nm laser excitation source over a wavenumber range of 50-3500 cm⁻¹.

Photoluminescence emission and excitation spectra were measured at room temperature with a FLS1000 fluorescence spectrometer (Edinburgh Instruments) using a 450 W continuous and a 450 W pulsed xenon lamp. Diffuse reflectance measurements were performed with a Shimadzu UV-3600 (Shimadzu Corporation, Tokyo, Japan), using BaSO_4_ white powder as the standard reference. Thermoluminescence (TL) spectra and long-persistent luminescence decay curves were obtained using a TOSL-24S instrument (RongFan, Guangzhou, China). The heating rates were set at 2 K/s for the preheating stage, 1 K/s for the measurement stage, and 5 K/s for the annealing stage. Prior to the measurements, all samples were subjected to a pre‑heating treatment to empty the internal electron traps. Spectral measurements were performed with a high-sensitivity CCD system covering 350-1000 nm, equipped with a single-photon PMT detector. Due to the temperature limitation of the current PL measurement system, temperature-dependent PL spectra above 500 K were not accessible in this work.

*DFT Calculation:* All structural relaxation and electronic property calculations were performed using the Vienna Ab Initio Simulation Package (VASP)^[1-2]^. The electron-electron interactions were treated using the Generalized Gradient Approximation (GGA)^[3]^, within the Perdew-Burke-Ernzerhof (PBE) functional framework^[3]^. The pseudopotentials used in these calculations were based on the Projector Augmented Wave (PAW) method^[4]^. The electronic wave function calculations employed a cutoff energy of 500 eV. During the structural relaxation, all atoms were allowed to relax fully until the residual force on each atom was minimized to below 0.05 eV/Å, ensuring the energy of the system was converged. The electronic energy relaxation process was carried out to a convergence criterion of 10⁻^5^ eV. For the Brillouin zone sampling^[5]^ in structural relaxation, a 1×1×2 k-point grid was used. Based on the optimized structures, the electronic band structures and density of states (DOS) for both LGO and LGO:Cu,Zn were calculated. Furthermore, the formation energies for various point defects, including oxygen vacancies (V_O_), lithium vacancies (V_Li_), gallium vacancies (V_Ga_), interstitial oxygen (O_i_), interstitial lithium (Li_i_), interstitial gallium (Ga_i_), and the antisite defect Li_Ga_/Ga_Li_, were systematically evaluated in both systems.

**Table S1.** Rietveld refinement results of the LGO and LGO:Cu,Zn samples.

| Phosphors | Cell parameters (Å) | Cell volume(Å³) | R_wp_ ,R_p_ ,χ^2^ |
| --- | --- | --- | --- |
| LGO | a = b = c = 8.19720 | 550.804 | R_wp_=0.0891  R_p_=0.0651  χ^2^=3.796 |
| LGO:Cu,Zn | a = b = c = 8.21323 | 554.040 | R_wp_=0.0514  R_p_=0.0336  χ^2^=1.89 |

**Figure S1.** (a) XRD patterns of LGO: xCu^2^(0.025%≤x≤0.25%) phosphors. (b) XRD patterns of LGO: 0.05%Cu^2^ yZn(0.05≤y≤0.5) phosphors.

**Figure S2.** Rietveld refinement of the typical XRD pattern.

**Figure S3.** Raman spectra of LGO, LGO:Cu, LGO:Zn, and LGO:Cu,Zn phosphors.

**Figure S4.** PersL decay curve of LGO: xCu^2+^(0.025% ≤ x ≤ 0.25%) samples (b) PersL decay curve of LGO: 0.05%Cu^2+^ xZn^2+^(0 ≤ x ≤ 50%) samples (c) TL spectra of the LGO: 0.05%Cu^2+^ xZn^2+^ (0 ≤ x ≤ 50%).

**Figure S5.** The excitation duration-dependent TL curves of LGO:Cu,Zn measured after excitation by 254 nm for various irradiation times (5 min - 35 min)

**Figure S6.** a,b) The temperature-dependent LPL decay curves from 300 to 600 K for LGO and LGO:Cu,Zn (pre-irradiated with UV light for 30 min)

**Figure S7.** Comparison of high-temperature persistent luminescence decay curves of LGO:Cu,Zn, ZGO:Cr, and LGO:Cr at 500 K.

**Figure S8.** Temperature-dependent normalized emission intensities of of LGO:Cr, ZGO:Cr and LGO:Cu,Zn under 254 nm excitation from 300 to 500 K.

**Figure S9.** Cyclic afterglow decay curves of LGO:Cu,Zn at 500 K over five cycles, showing good reproducibility

**Figure S10.** PL spectra of LGO and LGO:Cu,Zn

**Figure S11.** The temperature-dependent PL spectra under 254 nm excitation from 300 to 500 K for LGO:Cu,Zn.

**Figure S12.** a,b,c) Three consecutive heating–cooling cycles of the temperature-dependent normalized blue and red emission intensities of LGO:Cu,Zn.

**Figure S13.** The temperature-dependent XRD patterns of LGO:Cu,Zn.

**Figure S14** PL spectra of LGO, LGO:Cu,Zn (Air) and LGO:Cu,Zn (Ar) at different temperatures

**Figure S15. (a)** TL spectra of LGO and LGO:Cu,Zn sintered under air and Ar atmospheres (b) The LPL decay curves of LGO and LGO:Cu,Zn sintered under air and Ar atmospheres at 300 K (c) The LPL decay curves of LGO and LGO:Cu,Zn sintered under air and Ar atmospheres at 500 K (d) PersL emission spectra of LGO and LGO:Cu,Zn at 300 K

**Figure S16.** XPS O 1s spectra of LGO:Cu,Zn synthesized under reducing atmosphere

**Figure S17.** a,b) The UV DRS of LGO and LGO:Cu,Zn

**Figure S18.** The PDOS of LGO and LGO:Cu,Zn.

**Figure S19.** The band structure of LGO and LGO:Cu,Zn.

**Reference**

[1]	G. Kresse, J. Furthmüller, *Comput Mater Sci.* **1996**, 6, 15-50.

[2]	G. Kresse, J. Furthmüller, *Physical Review B.* **1996**, 54, 11169.

[3]	J.P. Perdew, K. Burke, M. Ernzerhof, *Physical Review Lett.* **1996**, 77, 3865-3868.

[4]	P. E. Blöchl, *Physical Review B*. **1994**, 50, 17953.

[5]	H. J. Monkhorst, J. D. Pack, *Physical Review B.* **1976**, 13, 5188.
